# Extracellular Vesicles Derived from Selenium-Deficient MAC-T Cells Aggravated Inflammation and Apoptosis by Triggering the Endoplasmic Reticulum (ER) Stress/PI3K-AKT-mTOR Pathway in Bovine Mammary Epithelial Cells

**DOI:** 10.3390/antiox12122077

**Published:** 2023-12-05

**Authors:** Yu Chen, Xiangqian Zhang, Jing Yang, Wen Feng, Ganzhen Deng, Shiwen Xu, Mengyao Guo

**Affiliations:** 1 College of Veterinary Medicine, Northeast Agricultural University, Harbin 150030, China; yuchen@hainanu.edu.cn (Y.C.); shiwenxu@neau.edu.cn (S.X.); 2 School of Tropical Agriculture and Forestry, Hainan University, Haikou 570228, China; 3 College of Veterinary Medicine, Huazhong Agricultural University, Wuhan 430070, China; zhangxiangqian215@webmail.hzau.edu.cn (X.Z.); jingyang@webmail.hzau.edu.cn (J.Y.); fengwen@webmail.hzau.edu.cn (W.F.); dgz@mail.hzau.edu.cn (G.D.)

**Keywords:** selenium deficiency, extracellular vesicles, endoplasmic reticulum stress, mastitis

## Abstract

Selenium (Se) deficiency disrupts intracellular REDOX homeostasis and severely deteriorates immune and anti-inflammatory function in high-yielding periparturient dairy cattle. To investigate the damage of extracellular vesicles derived from Se-deficient MAC-T cells (SeD-EV) on normal mammary epithelial cells, an in vitro model of Se deficiency was established. Se-deficient MAC-T cells produced many ROS, promoting apoptosis and the release of inflammatory factors. Extracellular vesicles were successfully isolated by ultrahigh-speed centrifugation and identified by transmission electron microscopy, particle size analysis, and surface markers (CD63, CD81, HSP70, and TSG101). RNA sequencing was performed on exosomal RNA. A total of 9393 lncRNAs and 63,155 mRNAs transcripts were identified in the SeC and SeD groups, respectively, of which 126 lncRNAs and 955 mRNAs were differentially expressed. Furthermore, SeD-EV promoted apoptosis of normal MAC-T cells by TUNEL analysis. SeD-EV significantly inhibited Bcl-2, while Bax and Cleaved Caspase3 were greatly increased. Antioxidant capacity (CAT, T-AOC, SOD, and GSH-Px) was inhibited in SeD-EV-treated MAC-T cells. Additionally, p-PERK, p-eIF2α, ATF4, CHOP, and XBP1 were all elevated in MAC-T cells supplemented with SeD-EV. In addition, p-PI3K, p-Akt, and p-mTOR were decreased strikingly by SeD-EV. In conclusion, SeD-EV caused oxidative stress, thus triggering apoptosis and inflammation through endoplasmic reticulum stress and the PI3K-Akt-mTOR signaling pathway, which contributed to explaining the mechanism of Se deficiency causing mastitis.

## 1. Introduction

Mastitis occurs frequently in the periparturient period of high-yielding cows [1]. During this period, excessive fat mobilization is mobilized to maintain successful lactation and combat negative energy balance, which may lead to overproduction of reactive oxygen species (ROS) [2,3]. Studies have confirmed that excessive ROS production could lead to oxidative stress, changing the immunity and anti-inflammatory function of perinatal cows, increasing their susceptibility to mastitis [4,5]. Oxidative stress in dairy cows with mastitis is caused by various factors including dramatic hormonal changes, exotic pathogen infections, and selenium (Se) deficiency [6,7]. Plasma glutathione peroxidase (GPx-3) plays a vital role in the antioxidant defense system of dairy cows [8]. GPx-3, a selenocysteine-containing extracellular antioxidant protein, is a crucial selenoprotein involved in the oxygen-free radical scavenging system [9]. Its function catalyzes the reduction of hydrogen peroxide and lipid hydroperoxides [10]. A low level of Se inhibited GPx activity to promote oxidative stress of the mammary gland, which was related to proliferation inhibition and apoptosis of mammary epithelial cells [11]. It has also been reported that the level of Se in cows is strongly correlated with the sensitivity of the mammary glands to bacteria. However, the exact mechanism of this antibacterial activity remains unclear [12,13]. Therefore, it is necessary to explore the role of selenium in the occurrence of mastitis.

Endoplasmic reticulum (ER) stress can also be triggered by Se deficiency-mediated oxidative stress and energy metabolism disorders. In normal physiology, ER homeostasis is maintained through BIP protein binding to ER stress sensors (ATF6, IRE1, and PERK) in a non-activated state bound by BIP [14,15]. When misfolded or unfolded proteins accumulate in the ER, ER stress sensors are activated after BIP release [16]. After PERK dimerization and phosphorylation activation, EIF2α protein phosphorylation results in the cessation of translation of most proteins and the subsequent cell growth cycle, inhibiting the ribosome from continuing to translate [17]. However, the translation of ATF4 is not inhibited, which enters the nucleus and induces CHOP protein leading to apoptosis [18]. When activated by IRE1, the XPB1 transcript is cleaved to form a new transcript and a new XBP1 protein inducing autophagy or apoptosis [19].

Bovine mammary epithelial cells are polar cells with vigorous secretory function. Extracellular vesicles are natural nanoparticles with diameters between 40 and 160 nm that play an essential role in intercellular communication [20]. Milk extracellular vesicles are absorbed by recipient cells through specific mechanisms to achieve selective cargo transport [21]. Lacto-extracellular vesicles have been reported in drug transport, health biomarkers, and epigenetic regulation [22,23,24,25]. With the rapid development of next-generation sequencing, many transcripts have been found in extracellular vesicles through RNA-sequencing technology (RNA-seq). Some of these transcripts are noncoding RNAs (ncRNAs) and may have diverse functions in biological processes [26]. Among them, lncRNAs are involved in chromatin modification, epigenetic regulation, genomic imprinting, transcriptional control, and pre-/post-translational mRNA processing. The correlations between lncRNAs and diverse mammal diseases have been clarified by many researchers, but the cognition about bovine mastitis-related lncRNAs remains limited. LncRNAs reported to be involved in mastitis were XIST [27], lncRNA-TUB [28], lncRNA H19 [29], LRRC75A-AS1 [30], and lnc-AFTR [31]. There are few reports of lncRNA associated with Se deficiency-induced diseases.

The cargoes of extracellular vesicles in mastitis (transcriptome and proteome) have been reported in depth [32,33,34]. Se is an essential element involved in the function of mammary epithelial cells [35]. Se deficiency impedes cellular homeostasis through oxidative stress. Few studies have reported the transcriptome of Se-deficient extracellular vesicles secreted by mammary epithelial cells and their communication to neighboring cells. Therefore, the model of Se-deficient mammary epithelial cells in vitro was established, and extracellular vesicles were successfully isolated, to explore the expression profile of extracellular vesicles secreted by Se-deficient mammary epithelial cells (SeD-EV) and further explore the effect of SeD-EV on normal MAC-T cells.

## 2. Materials and Methods

### 2.1. Cell Culture

The bovine mammary epithelial cell line (MAC-T) was acquired from Shanghai Shunran Biology. The cells were maintained in DME/F-12 (1:1) medium supplemented with 5 µg/mL of insulin, 200 mmol/L of L-glutamine, 5 µg/mL of transferrin, 1 µg/mL of hydrocortisone, 1 µg/mL of progesterone, 10 ng/mL of recombinant human epidermal growth factor, 100 U of penicillin–streptomycin solution and 5% fetal bovine serum with extracellular vesicle elimination (Hyclone, contains a very low level of Se-25 μg/dL) at 37 °C with a 5% CO_2_ incubator. The selenium content in the cells was depleted by continuous culture to the 8th generation under the above culture conditions. At passage 9, the serum supplement in the medium was changed to 2%. The supplemented medium with 10 ng/mL of selenomethionine (SeMet) was defined as the selenium control group (SeC), while the addition of no SeMet was defined as the selenium-deficient group (SeD). The cell supernatant was harvested to extract extracellular vesicles.

### 2.2. Detection of Intracellular Se Levels

Se concentration was detected by fluorescence spectrophotometry. In short, cells were digested by pancreatic enzymes and collected for counting. The cells obtained after centrifugation were precipitated under ultrasonic treatment to crack the cell membrane. After digestion by nitric acid and perchloric acid (3:2) and reduction by hydrochloric acid, the multifunctional enzyme spectrometer was used to determine the absorbance at Ex = 376.3 nm/Em = 520 nm.

### 2.3. ROS Analysis

The DHE-ROS Detection Kit was obtained from BestBio (Shanghai, China). The ROS level was examined to follow the manufacturer’s instructions. The DHE probe was diluted to working fluid with DME/F-12 medium, and the cells were incubated with the DHE probe for 30 min at 37 °C. The fluorescence microscope was used to observe the results at Ex = 535 nm/Em = 610 nm.

### 2.4. Evaluation of Antioxidant Biochemical Indexes

A catalase (CAT) detection kit (visible light), malondialdehyde (MDA) detection kit (TBA method), total antioxidant capacity (T-AOC) detection kit, glutathione peroxidase (GSH-Px) detection kit, and superoxide dismutase (SOD) detection kit (WST-1 method) were brought from Nanjing Jiancheng Bioengineering Institute. The collected cells were lysed using an ultrasonic crusher. The BCA protein quantification kit was used to measure the protein concentration. BCA CAT, MDA, T-AOC, SOD, and GSH-Px were measured according to the manufacturer’s instructions.

### 2.5. ELISA

Proinflammatory cytokines were detected with ELISA kits (BioLegend, San Diego, CA, USA) following the manufacturer’s manuals. The sample concentration value was calculated according to the standard curve. At the same time, the protein concentration of each sample was measured by the BCA method. Finally, the protein concentration of each sample was used to correct for TNF-α, IL-6, and IL-1β concentrations in the sample, and the results were expressed as “ng/mg protein”.

### 2.6. TUNEL Assay

MAC-T cells of different groups were prepared into cell slides and fixed at 4 °C for 1 h with 4% paraformaldehyde. Cell slides were permeated with 0.2% Triton X-100. After balancing, the mixture of TdT and dUTP (1:9) was incubated at 37 °C for 1 h and then stained with DAPI in the dark for 10 min. Apoptosis was observed by fluorescence microscope.

### 2.7. Isolation of Extracellular Vesicles

The collected cell supernatants were centrifuged by gradients of 300× *g*, 2000× *g*, and 18,000× *g*, respectively. The ultrahigh-speed centrifuges centrifuged the liquid twice at 110,000× *g* speed at 4 °C for 70 min. The white precipitate was collected for further study.

### 2.8. TEM

Extracellular vesicles were added to a clean copper mesh with a carbon-coated Formvar support membrane. The filter paper was used to absorb the liquid after 10 min slowly. The copper mesh was then treated with 2.5% glutaraldehyde, 40 g/L uranium dioxyacetate, and 10 g/L methylcellulose. After natural drying, TEM was used to observe the result at 80 kV.

### 2.9. Particle Size Analysis

Particle size distribution of extracellular vesicles was assessed with the ZETASIZER Nanoparticle Tracking Analysis (NTA) instrument. Parameters of the instrument were set to Electrolyte: PBS; Temperature: 25 °C sensed; pH 7.0 entered; Conductivity: 15,000.00 µS/cm sensed.

### 2.10. RNA Sequencing and Analysis

Exosomal RNA was purified by a TIANSeq rRNA Depletion Kit. cDNA libraries were constructed by reverse transcription, terminal repair and splicing, adapter ligation, PCR amplification, post-amplification clean-up, and library quality control. After library quality inspection, sequencing was performed on the Illumina HiSeq 4000 platform. Raw data were processed with data filtering, decoupling sequences, and removing low-quality reads. For the detected mRNAs, the gene expression levels were calculated and the differentially expressed genes among the samples were analyzed. The differentially expressed genes were analyzed by GO and KEGG analysis. For the detected lncRNAs, the differentially expressed lncRNAs among samples were analyzed. Different lncRNAs were selected for target gene prediction to explore the potential functions of lncRNAs.

### 2.11. RT-qPCR

TRIzol reagent was employed to isolate the total RNA of extracellular vesicles and cells. RNA was reverse-transcribed into cDNA using the 1st Strand cDNA Synthesis SuperMix (Yeasen, Shanghai, China). RT-qPCR was performed with SYBR Green qPCR Master Mix (Yeasen, Shanghai, China) following the manufacturer’s instructions. Internal reference gene GAPDH normalized the relative mRNA expression with the 2^−△△Ct^ method. The primers used for RT-qPCR are listed in Table 1.

### 2.12. Western Blot

Total cell protein was harvested through RIPA lysate. The concentration of each protein sample was measured with the BCA protein quantification kit. Each sample with an equal amount of protein was separated with 10% SDS-PAGE. Then, the separated protein was transferred onto PVDF membranes at 200 mA. After being blocked with blocking buffer, the membranes were incubated with primary antibodies overnight at 4 °C. Membranes were washed and incubated with secondary antibodies and visualized using enhanced chemiluminescence.

### 2.13. Extracellular Vesicles Tracing

A PKH67 Green Fluorescent Cell Linker Mini Kit (Sigma-Aldrich, MINI67, St. Louis, MO, USA) was used to label isolated extracellular vesicles according to the manufacturer’s instructions. Briefly, PKH67 was diluted into working fluid and added to extracellular vesicles for staining for 10 min. The PKH67-labeled extracellular vesicles were then obtained by ultrahigh-speed centrifugation to remove the excess dye. The cells were co-cultivated with PKH67-labeled extracellular vesicles for 6 h, then the cells were stained with DAPI and observed with a laser confocal microscope.

### 2.14. Statistical Analysis

GraphPad Prism 5.0 software was used for statistical analysis. Data were expressed as the mean ± standard for 3 independent experiments. Student’s *t*-test or one-way analysis of variance (ANOVA) was used to compare the two groups’ differences.

## 3. Results

### 3.1. Se Deficiency Promoted MAC-T Cell Damage through Oxidative Stress

**Figure 1:**
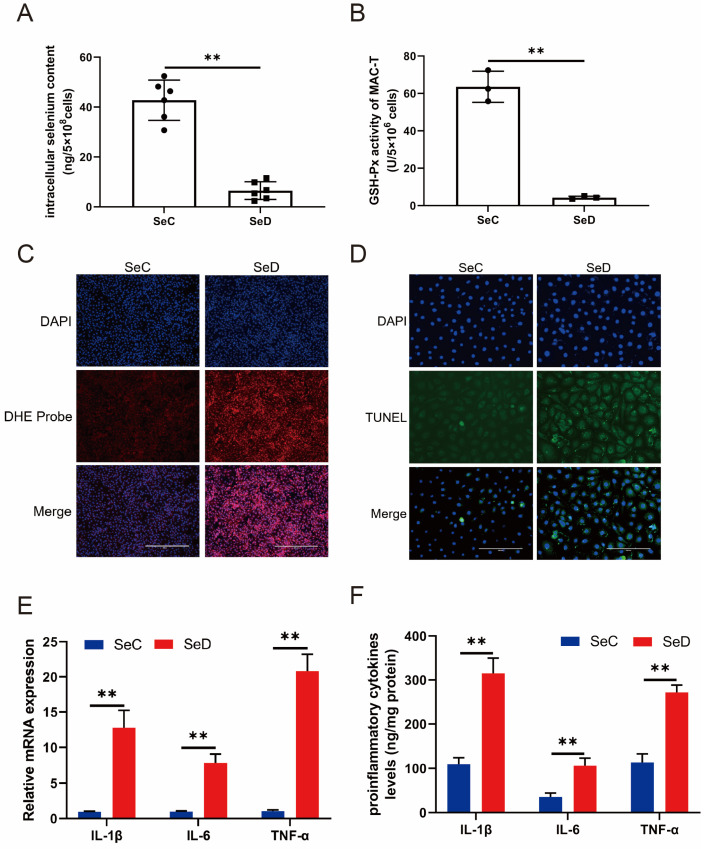
Se deficiency promoted MAC-T cell damage through oxidative stress. (**A**) The intracellular Se concentration was measured using fluorescence spectrophotometry. (**B**) The activity of GSH-Px was detected with a GSH-Px assay kit. (**C**) The ROS level was measured by fluorescence intensity using a DHE probe. The scale bar = 400 µm. (**D**) TUNEL analysis was used to assess apoptosis in Se-deficient MAC-T cells. The scale bar = 200 µm. (**E**) The Se-deficient cells were supplemented with/without SeMet, and the level of TNF-α, IL-6, and IL-1β mRNA expression was measured by qPCR. (**F**) ELISA was employed to test the pro-inflammatory factors. ** *p* < 0.01.

Fluorescence spectrophotometry was used to determine the cell Se level, and it was found that the intracellular Se content in the SeD group was much lower than that in the SeC group (Figure 1A), indicating that the cell model of Se deficiency was successfully constructed. GSH-Px is a peroxide-decomposing enzyme containing Se, and its activity can reflect the level of Se in the body. Compared with the SeC group, the activity of GSH-Px in the SeD group was significantly decreased (Figure 1B), suggesting that the lack of Se inhibited the free radical scavenging system. Meanwhile, DHE probe was performed to display ROS levels by fluorescence intensity, and it was revealed that the ROS level in the SeD group was higher than those in the SeC group (Figure 1C). In addition, TUNEL analysis showed that Se deficiency-induced apoptosis occurred in MAC-T cells (Figure 1D). The mRNA levels of proinflammatory cytokines were detected by RT-qPCR, and the relative mRNA levels of proinflammatory cytokines were significantly increased in Se-deficient MAC-T cells (Figure 1E). At the same time, cytokine protein levels were also evaluated by ELISA, and the trend of ELISA results was found to be consistent with RT-qPCR results (Figure 1F).

### 3.2. Identification of Extracellular Vesicles

**Figure 2:**
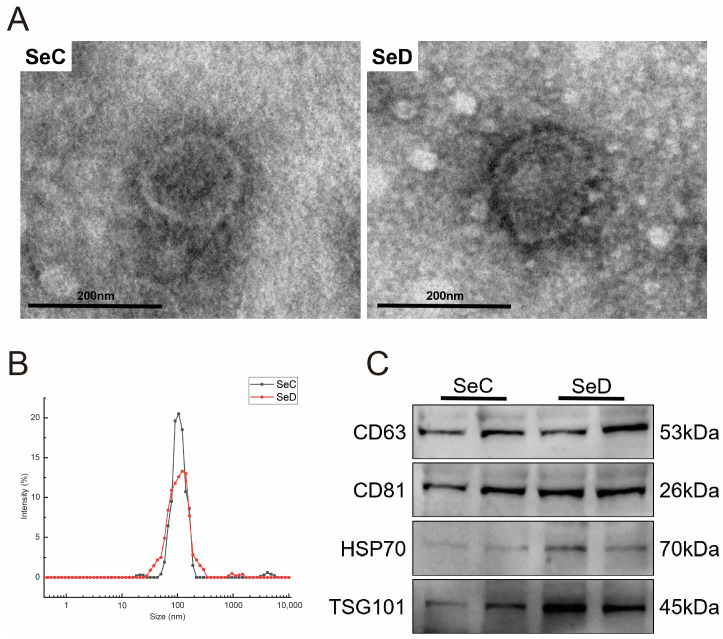
Identification of extracellular vesicles. (**A**) TEM was used to observe the morphology of extracellular vesicles. (**B**) Particle size analysis of extracellular vesicles. (**C**) Extracellular vesicle biomarkers (CD63, CD81, HSP70, and TSG101) were examined using Western blot.

TEM was employed to observe exosome morphology. The isolated extracellular vesicles showed a pronounced monolayer membrane structure and saucer-like shape under electron microscopy (Figure 2A). NTA analysis was used to evaluate the size distribution of extracellular vesicles. As shown in Figure 2B, the main peak of the particle size of isolated extracellular vesicles was about 100 nm, and more than 90% of the extracellular vesicles were concentrated between 70 and 150 nm. In addition, extracellular vesicle surface characteristic markers were detected with Western blot. The results showed that CD63, CD81, HSP70, and TSG101 were detected successfully (Figure 2C).

### 3.3. Overview of RNA Sequencing

**Figure 3:**
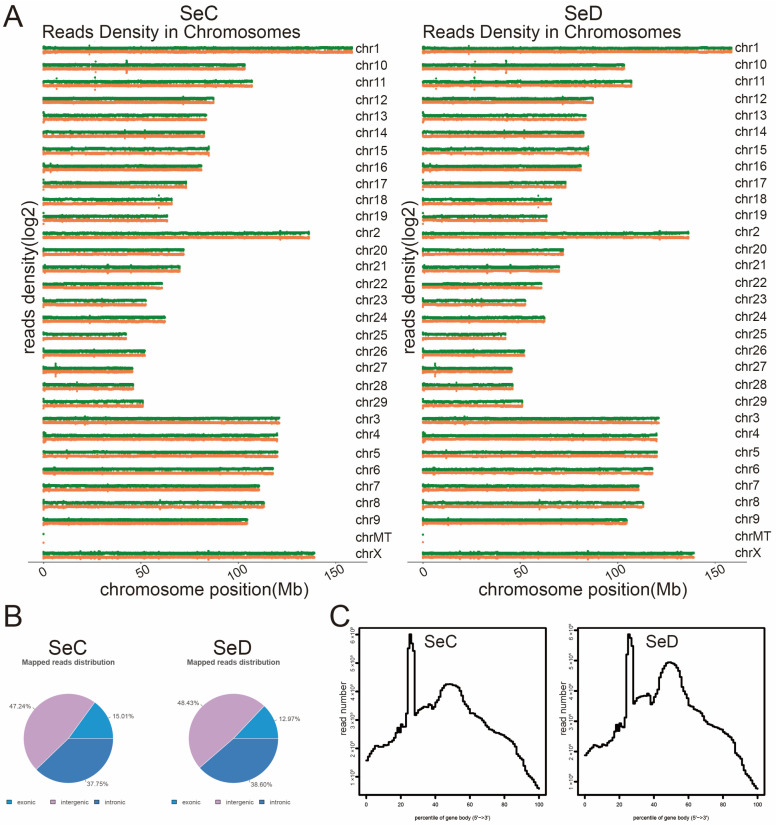
Analysis of reads mapping to the reference genome. (**A**) Distribution of reads on chromosomes. The horizontal axis represents the location of chromosomes, and the vertical axis indicates the abundance of reads. The higher the peak value, the more reads detected there. Green is the plus chain. Yellow is the negative chain. (**B**) Distribution of reads among genomic components. (**C**) Transcript coverage uniformity. The X-coordinate indicates gene length (in percentage), and the Y-coordinate indicates the number of reads in the region.

In this study, four cDNA libraries were constructed using exosomal RNA isolated from two SeC groups and two SeD groups. Additionally, 241,811,846 raw reads of 150 bp were produced from the four cDNA libraries (Table 2), and 237,947,153 clean reads were obtained after data filtering (Table 3). The mapped clean reads in four groups were more than 78%. The uniquely and multiple-mapped clean reads were more than 73% and 5%, respectively. Approximately 39% of reads map primarily to the “+” and “−” chains of the genome (Table 4). After genome alignment was completed, the location information of all reads’ corresponding genomes was counted to evaluate the coverage depth of sequencing data. As shown in Figure 3A, reads were randomly distributed on the 31 chromosomes of Bos taurus. Subsequently, the reads mapping on chromosomes were annotated on exonic, intronic, and intergenic. Reads distribution statistics are displayed in Figure 3B. In addition, the uniformity of transcript coverage is shown in Figure 3C.

### 3.4. Differential Expression of mRNA

**Figure 4:**
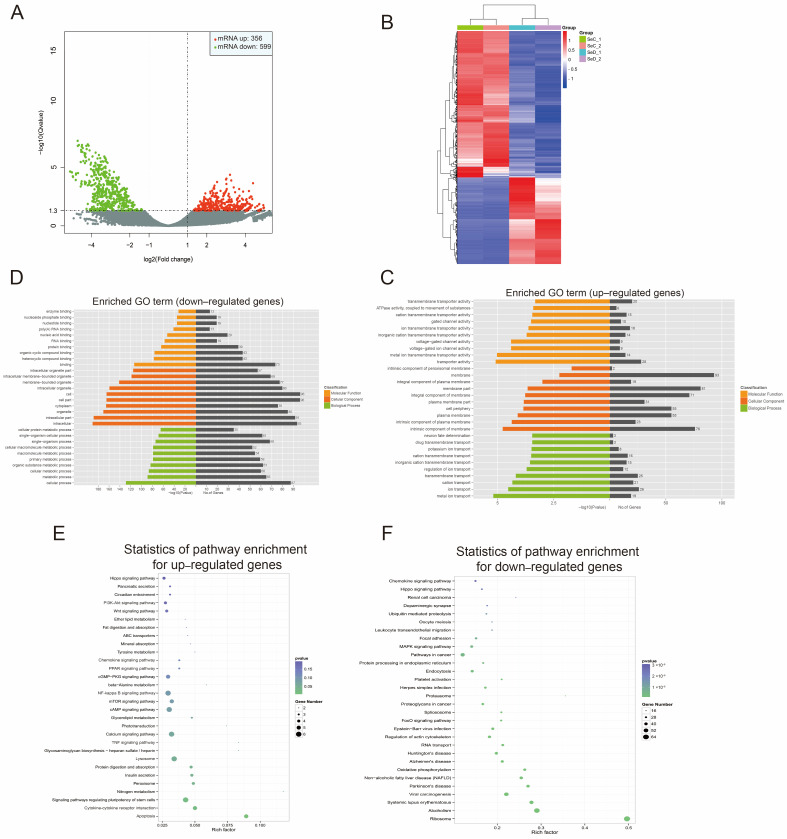
Differential expression of mRNA. (**A**) Volcano plot of differentially expressed mRNAs. (**B**) Clustering of differential expression genes. (**C**) GO analysis on upregulated genes. (**D**) GO enrichment on downregulated genes. (**E**) KEGG analysis for upregulated genes. (**F**) KEGG analysis for downregulated genes.

RNA-seq yielded 63,155 mRNA transcripts after a stringent filtering process. The differentially expressed mRNAs were screened, 356 were upregulated, and 599 were downregulated (Figure 4A). As illustrated in Figure 4B, differentially expressed mRNAs were analyzed by hierarchical clustering. The differentially expressed mRNAs were further annotated by GO analysis. Upregulated mRNAs were primarily focused on metal ion/inorganic ion transport, plasma membrane/peroxisome membrane, etc. (Figure 4C). The downregulated mRNAs were mainly enriched in the metabolic process (organic substance metabolic process, primary metabolic process, macromolecule metabolic process), binding (heterocyclic compound binding, organic cyclic compound binding, protein binding, RNA binding), cellular response (cellular response to hydrogen peroxide, cellular response to oxidative stress, response to oxidative stress), etc. (Figure 4D). KEGG pathway enrichment revealed that upregulated mRNAs were highly enrolled in apoptosis, peroxisome, lysosome, PI3K-Akt, mTOR, and PPRG signaling pathways (Figure 4E). In contrast, downregulated mRNAs involved specifically include ribosome, oxidative phosphorylation, MAPK signaling pathway, FoxO signaling pathway, and endocytosis (Figure 4F).

### 3.5. Differential Expression of lncRNA

**Figure 5:**
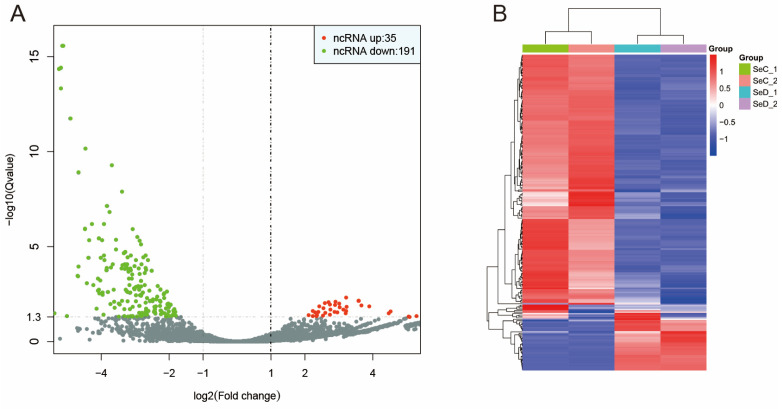
Differential expression of lncRNA. (**A**) Volcano plot of differential expression of lncRNA. (**B**) Clustering of differentially expressed lncRNA.

A total of 9393 lncRNAs were identified through the characterization of lncRNA. Among them, 35 upregulated and 191 downregulated lncRNAs were found (Figure 5A). The differentially expressed lncRNAs are also clustered in Figure 5B.

### 3.6. Prediction of lncRNA Target Genes

**Figure 6:**
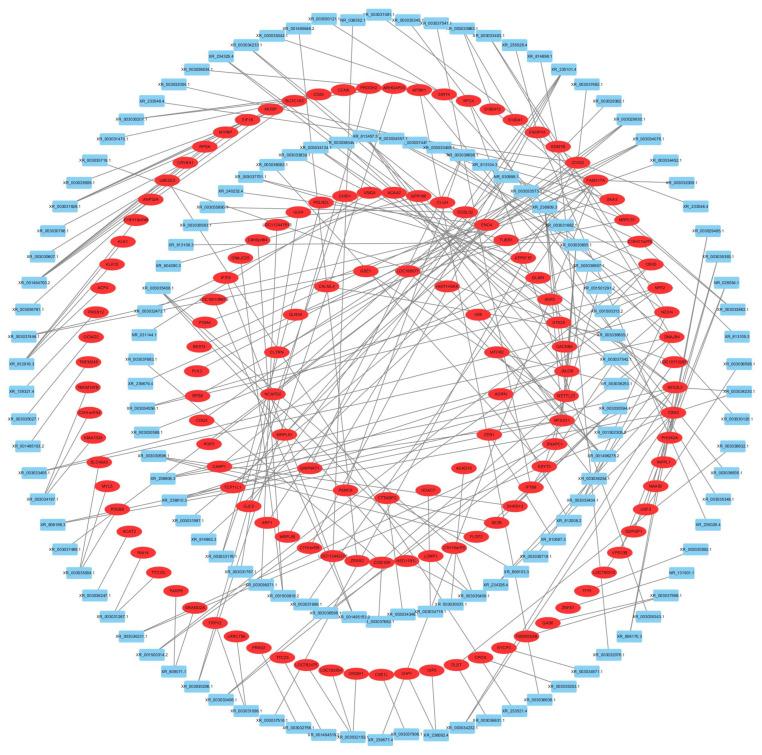
lncRNA regulation of target genes in Cis. The blue represents lncRNA, and the red indicates target gene.

lncRNA regulation of target genes can be predicted with two patterns: In Cis regulation and In Trans regulation. The target genes of lncRNA In Cis are shown in Figure 6.

### 3.7. GO and KEGG Analysis of lncRNA Target Genes

**Figure 7:**
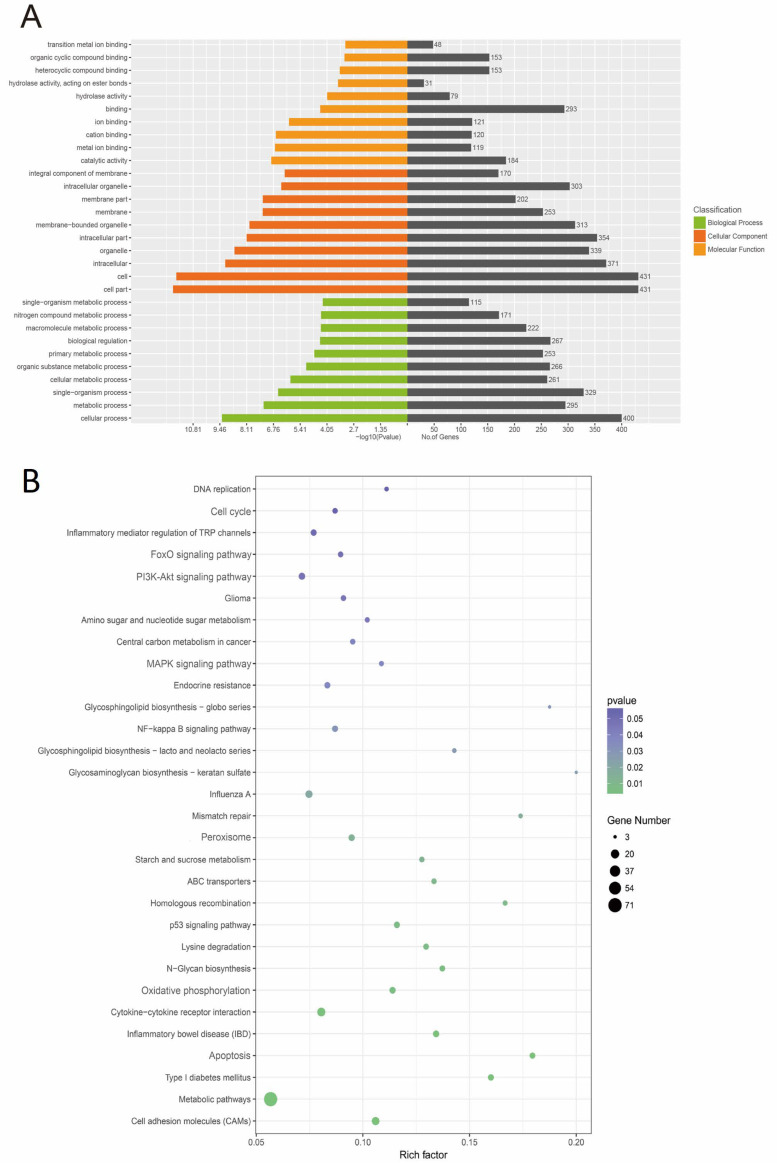
GO and KEGG analysis of lncRNA target genes. (**A**) GO enrichment analysis on lncRNA target genes. (**B**) The KEGG pathway enrichment analysis on lncRNA target genes.

Next, the target genes of lncRNA were further analyzed through GO enrichment (Figure 7A). GO in BP term illustrated that they were primarily enriched in response to oxidative stress and cellular response to hydrogen peroxide. CC term was closely related to the membrane part (cytoplasm and peroxisome membrane). MF term mainly involved protein binding, metal ion binding, and hydrolase activity. Most of the KEGG pathway was enriched in apoptosis, peroxisome, oxidative phosphorylation, p53 signaling pathway, MAPK signaling pathway, PI3K-Akt signaling pathway, NF-kappa B signaling pathway, FoxO signaling pathway, and so on (Figure 7B).

### 3.8. SeD-EV Induced Apoptosis and Inflammation of Normal MAC-T Cells

**Figure 8:**
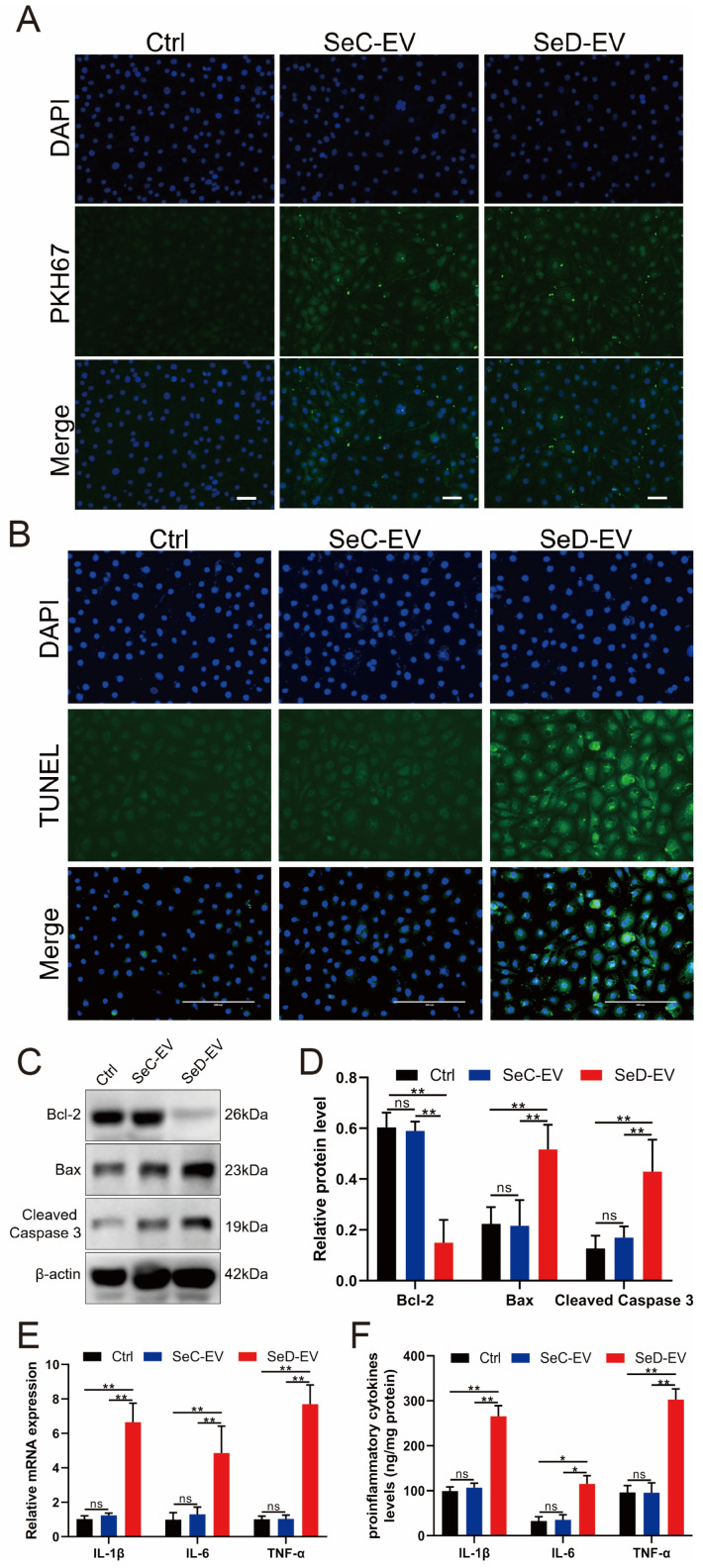
SeD-EV induced apoptosis and inflammation of normal MAC-T cells. (**A**) SeC-EV and SeD-EV were taken up by MAC-T cells and traced by PKH67 dye. Scale bar = 50 μm. (**B**) TUNEL analysis was employed to evaluate apoptosis in Se-EV-treated MAC-T cells. Scale bar = 200 μm. (**C**) Apoptosis-associated proteins (Bcl-2, Bax, and Cleaved Caspase3) were evaluated by Western blot. (**D**) Relative protein expression levels were quantified using ImageJ software (v1.8.0). (**E**) The relative expression levels of TNF-α, IL-6, and IL-1β mRNA were detected by qPCR. (**F**) ELISA was used to measure the level of pro-inflammatory factors. * *p* < 0.05; ** *p* < 0.01; ns: not significant.

To further explore the effects of SeD-EV on normal MAC-T cells, SeD-EV was added to a normal cell culture medium. The isolated extracellular vesicles were labeled with PKH67 dye to confirm cell uptake. As illustrated in Figure 8A, green fluorescent extracellular vesicles were taken into the cell under a fluorescence microscope. SeD-EV promoted apoptosis of normal MAC-T cells by TUNEL analysis (Figure 8B). Furthermore, apoptosis-associated proteins were evaluated by Western blot, which revealed that SeD-EV significantly inhibited the anti-apoptotic protein Bcl-2, while Bax and Cleaved Caspase3 were greatly increased (Figure 8C,D). As for the inflammatory response, proinflammatory factors were examined with RT-qPCR and ELISA, and they all displayed significant upregulation of cytokine expression at mRNA and protein levels on SeD-EV-treated MAC-T cells (Figure 8E,F).

### 3.9. SeD-EV Promoted ER Stress by Oxidative Stress

**Figure 9:**
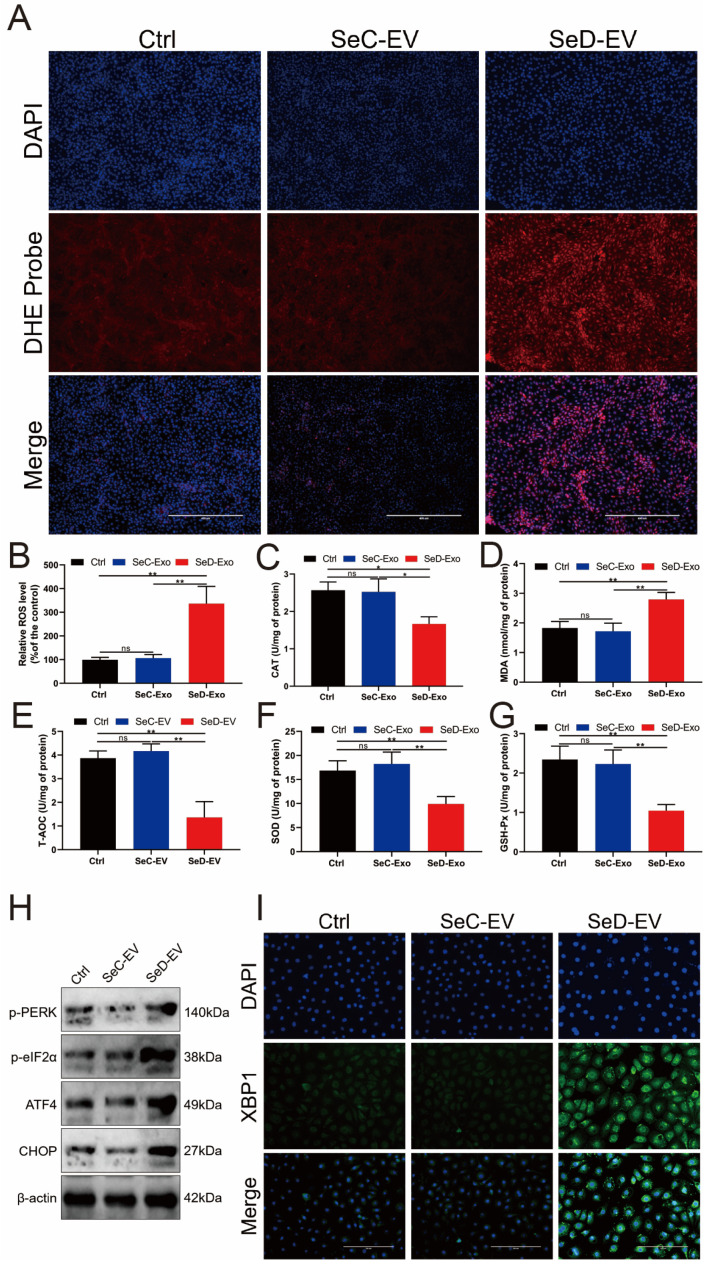
SeD-EV promoted ER stress by oxidative stress. (**A**) SeD-EV was co-cultured with MAC-T cells, and the ROS level was assessed. Scale bar = 400 μm. (**B**) Relative fluorescence intensity was quantified using ImageJ software. (**C**) The activity of CAT. (**D**) The concentration of MDA. (**E**) The level of T-AOC. (**F**) The activity of SOD. (**G**) The activity of GSH-PX. (**H**) CHOP, p-eIF2α, ATF4, and p-PERK were elevated with immunoblotting in SeD-EV-treated MAC-T cells. (**I**) XBP1 protein was assessed by indirect immunofluorescence. Scale bar = 200 μm. * *p* < 0.05; ** *p* < 0.01; ns: not significant.

Based on bioinformatics analysis of the above differential genes, we found that the part of the differential genes in the expression profile of SeD-EV was closely related to oxidative stress and ER stress. Thus, antioxidant capacity was evaluated in SeD-EV-treated MAC-T cells. The ROS assessment found that SeD-EV supplementation of MAC-T cells produced dramatically a large number of red fluorescence signals (Figure 9A), and the relative quantification of fluorescence intensity by ImageJ software found that the ROS level in the SeD group was much more than that in the SeC group (Figure 9B). Compared with the SeC-EV group, catalase (CAT) activity was significantly inhibited in the SeD-EV group (Figure 9C). In contrast, SeD-EV induced the production of more MDA in MAC-T cells (Figure 9D). T-AOC, SOD, and GSH-Px activity were all severely suppressed in MAC-T cells supplemented with SeD-EV (Figure 9E–G). Additionally, ER stress-associated proteins were measured by immunoblotting. p-PERK, p-eIF2α, ATF4, and CHOP were all elevated in SeD-EV-treated MAC-T cells (Figure 9H). XBP1 was evaluated by indirect immunofluorescence, and it was found that SeD-EV could significantly increase the expression of XBP1 (Figure 9I).

### 3.10. SeD-EV Inhibited PI3K-AkT-mTOR Signaling Pathways

**Figure 10:**
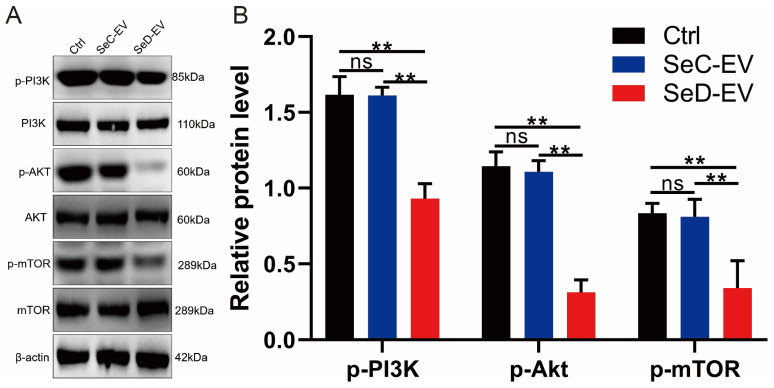
SeD-EV inhibited PI3K-AkT-mTOR signaling pathways. (**A**) PI3K, Akt, mTOR, and their phosphorylation form were determined with Western blot. (**B**) Relative protein expression levels were quantified using ImageJ software. ** *p* < 0.01; ns: not significant.

Western blot was used to evaluate the PI3K-Akt pathway-related proteins, and it was found that SeD-EV could inactivate PI3K, Akt, and mTOR to decrease their phosphorylation form (Figure 10A,B).

## 4. Discussion

Se is a vital essential trace element in animals, and selenoproteins are involved in the composition of the body’s antioxidant system [36]. Se deficiency destroys the body’s REDOX homeostasis, and ROS accumulation promotes the occurrence of oxidative stress in mastitis [37]. Previous studies have confirmed that Se deficiency causes inflammatory damage through oxidative stress in chickens [38], mice [39], carps [40], and cattle [41]. The present study established the Se-deficient MAC-T cell model in vitro by continuous delivery culture using FBS with very low Se concentrations. We found that Se deficiency decreased ROS clearance (GSH-Px), and intracellular ROS accumulation induced inflammation and apoptosis. These results were consistent with previous studies. In addition, extracellular vesicles derived from selenium-deficient MAC-T cells were successfully isolated and identified. The extracellular vesicles’ morphology, size, and markers are consistent with previous reports [42,43]. Extracellular vesicles act as a bridge of cell–cell communication, which has the potential to enter the cell in various ways and release their contents to transmit information [44,45]. In this study, after treating normal MAC-T cells with SeD-EV, SeD-EV was taken up into the cells and triggered inflammatory response and apoptosis. This suggested that SeD-EV cargo could trigger signal transduction in normal cells, and the effect was similar to that of Se deficiency treatment.

To explore transcriptome information in SeD-EV, RNA-seq was performed to build lncRNA and mRNA expression profiles in extracellular vesicles derived from Se-deficient MAC-T cells. We investigated the differential expression levels of lncRNAs and mRNAs between the SeC-EV group and the SeD-EV group, and a significant differential expression of 126 lncRNAs and 955 mRNAs was found. GO and KEGG analysis showed that differentially expressed mRNAs and lncRNA target genes were mainly involved in oxidative stress, oxidative phosphorylation, endocytosis, apoptosis, metabolism, and other processes. The transcriptional expression data of the liver in rats with an Se deficiency diet indicated that Se deficiency changed some biological states or processes, including oxidative phosphorylation, ROS, xenobiotic metabolism, fatty acid metabolism, and glutathione metabolism [46]. Other studies have shown that dietary Se consistently affects inflammatory or immune pathway signaling networks in liver and lung tissue in mice [47]. Our results and previous studies indicated that Se deficiency unbalanced the body’s REDOX system and promoted oxidative stress. As for how lncRNAs in SeD-EV play a regulatory role in oxidative stress, it is worthy of further investigation.

Recent studies have shown that more than half of selenoproteins are present in the ER, where they are involved in the maintenance of ER homeostasis, ER stress response signaling pathways, REDOX balance, glycoprotein folding and quality control, and Ca2+ homeostasis [48]. Therefore, Se deficiency can easily contribute to ER stress [49]. In our study, SeD-EV could promote the phosphorylation of PERK and eIF2α, increasing ATF4 and CHOP proteins to induce apoptosis. Furthermore, the expression of XBP1 obviously went up. The above indicated that SeD-EV promoted apoptosis of MAC-T cells through ER stress. The PI3K-AKT signaling pathway is one of the classic signal transduction pathways regulated by oxidative stress [50]. In this study, differentially expressed mRNAs were partially enriched in PI3K-AKT and mTOR signaling pathways in KEGG analysis. Interestingly, SeD-EV decreased the expression of p-PI3K and p-AKT in MAC-T cells, thereby inhibiting the activation of the PI3K-AKT pathway. Its downstream proteins, including Bax and Cleaved Caspase3, increased significantly. Since Bax and Cleaved Caspase3 induced apoptosis, SeD-EV promoted apoptosis and Se deficiency. A previous study reported that dietary Se deficiency and excess impaired PI3K-AKT signaling activation promote apoptosis in the testis [51]. In addition, it is worth noting that mTOR is another response element downstream of the PI3K-AKT pathway [52]. mTOR is an important regulator of cell growth and proliferation [53]. p-mTOR was suppressed by SeD-EV to inhibit cell proliferation. These results suggested that SeD-EV promoted inflammation and apoptosis via the PI3K-AKT-mTOR pathway.

## 5. Conclusions

**Figure 11:**
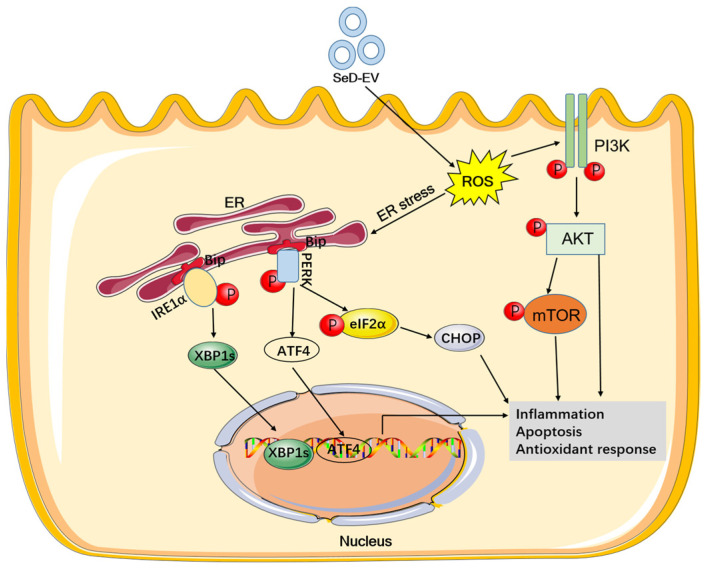
SeD-EV promoted apoptosis and inflammation through ER stress and the PI3K-AKT-mTOR pathway.

In summary, the results of this study suggest that extracellular vesicles derived from selenium-deficient MAC-T cells contained differentially expressed 126 lncRNAs and 955 mRNAs. SeD-EV and Se deficiency similarly exacerbated apoptosis and inflammation through ER stress and the PI3K-AKT-mTOR pathway (Figure 11). Our findings highlight the critical role of SeD-EV in Se-deficient mastitis. They provide new insights into the role of trace element Se in the occurrence of mastitis in dairy cows.

## Figures and Tables

**Table 1:** Sequence of primers for qPCR.

Gene	Primer Sequence (5′–3′)	NCBI Reference Sequence	Product Size
TNF-α	Sense: ACGGGCTTTACCTCATCTACTCA Anti-sense: GGCTCTTGATGGCAGACAGG	NM_173966.3	141 bp
IL-6	Sense: ATGCTTCCAATCTGGGTTCA Anti-sense: GAGGATAATCTTTGCGTTCTTT	NM_173923.2	268 bp
IL-1β	Sense: GGCAACCGTACCTGAACCCA Anti-sense: CCACGATGACCGACACCACC	NM_174093.1	206 bp
GAPDH	Sense: TGCTGGTGCTGAGTATGTGGTG Anti-sense: CAGTCTTCTGGGTGGCAGTGAT	NM_001034034.2	296 bp

**Table 2:** Summary of raw data quality control.

Sample	Total Reads	Total Reads (bp)	GC%	Error Rate (%)	Q20 (%)	Q30 (%)
SeC_1	61,456,724	9,135,825,342	49.01	0.12	98.82	96.45
SeC_2	60,867,218	9,057,475,381	48.93	0.11	98.63	96.28
SeD_1	59,355,422	8,903,313,300	49.35	0.12	98.57	95.68
SeD_2	60,132,482	8,998,594,475	49.99	0.13	98.43	95.59

**Table 3:** Statistics of filter data.

Sample	Raw Reads	Raw Bases	Clean Reads	GC%	Q20 (%)	Q30 (%)	Clean Bases	Clean Bases%
SeC_1	61,456,724	9,135,825,342	60,102,541	50.02	99.17	97.50	8,403,132,152	91.98
SeC_2	60,867,218	9,057,475,381	59,987,628	49.93	99.12	97.45	8,271,286,526	91.32
SeD_1	59,355,422	8,903,313,300	58,032,752	49.86	99.15	97.33	8,095,777,614	90.93
SeD_2	60,132,482	8,998,594,475	59,824,232	49.91	99.20	97.36	8,344,396,659	92.73

**Table 4:** Reads mapping analysis.

Sample	SeC_1	SeC_2	SeD_1	SeD_2
The effective reads	110,240,527 (100%)	109,856,243 (100%)	99,225,814 (100%)	102,453,768 (100%)
Total mapped	87,839,652 (79.68%)	86,654,605 (78.88%)	77,776,870 (78.38%)	800,368,834 (78.12%)
Multiple mapped	5,975,037 (5.42%)	5,888,295 (5.36%)	5,336,626 (5.38%)	5,399,313 (5.27%)
Uniquely mapped	81,853,591 (74.25%)	81,469,390 (74.16%)	72,440,244 (73.01%)	75,037,139 (73.24%)
Read1 mapped	44,482,053 (40.35%)	44,184,181 (40.22%)	38,935,504 (39.24%)	40,295,067 (39.33%)
Read2 mapped	44,360,788 (40.24%)	44,118,267 (40.16%)	38,841,366 (39.14%)	40,233,595 (39.27%)
Reads map to ‘+’	44,404,883 (40.28%)	44,162,210 (40.20%)	38,941,441 (39.25%)	40,315,557 (39.35%)
Reads map to ‘−’	44,228,499 (40.12%)	44,041,368 (40.09%)	38,835,429 (39.14%)	40,161,877 (39.20%)
Reads mapped in proper pairs	82,526,059 (74.86%)	82,018,671 (74.66%)	73,512,266 (74.09%)	76,369,038 (74.54%)

## Data Availability

Data are contained within the article.
